# Building bigger beta-barrels

**DOI:** 10.7554/eLife.44076

**Published:** 2019-01-21

**Authors:** Vikas Nanda

**Affiliations:** Center for Advanced Biotechnology and MedicineRutgers, The State University of New JerseyPiscatawayUnited States

**Keywords:** outer membrane proteins, beta barrels, repeat proteins, beta hairpin, outer membrane beta barrels, evolution, *E. coli*, Other

## Abstract

The range of barrel-shaped proteins found in the outer membrane of certain bacteria evolved through multiple pathways.

**Related research article** Franklin MW, Nepomnyachyi S, Feehan R, Ben-Tal N, Kolodny R, Slusky JSG. 2018. Evolutionary pathways of repeat protein topology in bacterial outer membrane proteins. *eLife* **7**:e40308. doi: 10.7554/eLife.40308

You would have a difficult time today if you were looking for a cooper to make you a bespoke wooden barrel. But if you were lucky enough to find one, you would likely tell them the desired height, girth and shape of your new barrel. They would then disappear into a workshop and painstakingly create a series of long wooden boards to serve as the staves – each with precise dimensions, curvature and beveling of edges to meet your specifications – and a few days later you would be the proud owner of a new, one-of-a-kind wooden barrel.

While coopering is an ancient trade with a thousand years of history, nature has been building barrel-shaped proteins for even longer. Today, as protein engineering advances to the point where we hope to build synthetic, bespoke molecular barrels for a range of applications, it will be essential to understand how nature evolved barrels and learn the tricks of the trade.

Building barrels from wood or from amino acids presents different challenges. Let us say that you find your barrel is too small and you would like to make the opening at the top wider. You would be dismayed to learn that because the dimensions of the wooden staves are unique to that design, your cooper cannot simply add more staves to make the opening wider – a new barrel would need to be built. In contrast, biology creates new protein forms by tweaking existing ones using the tools of genetic variation: mutation, duplication and recombination.

Barrel-shaped proteins called OMBBs (which is short for outer-membrane beta-barrels) are found in the outer membranes of gram-negative bacteria, with beta strands playing the role of staves. These OMBBs always contain an even number of strands, with each pair adding a fraction of a nanometer to the diameter of the barrel opening: the smallest OMBBs have just eight beta strands and the largest we know of contain 26 (Franklin et al., 2018b). By varying the number of beta strands they have, natural OMBBs can dictate the transit of proteins and molecules through them based on size. The ability to similarly adjust the dimensions of synthetic barrels by adding or removing strands would enable researchers to design and build structures called nanopores that could, for example, be used to sequence DNA, or to sense chemicals in the environment with high selectivity and sensitivity (Trick et al., 2014).

OMBBs are repeat proteins – a diverse class of proteins that consist of two or more copies of a simpler structural unit, which is thought to be a 'beta hairpin' (that is, a structure in which two beta strands are joined together at one end to create a structure shaped like a hairpin; Remmert et al., 2010). The repetitive nature of these structures, combined with the exclusion of water in the membrane in which they are embedded, reduces the complexity of their amino acid sequences. While less complexity may sound like a good thing, it makes it more difficult to use phylogenetic methods to unravel the evolutionary histories and relationships of the beta-barrels found in nature today. Now, in eLife, Joanna Slusky of the University of Kansas and colleagues – including Meghan Franklin as first author – report how they have combined sequence and structural information on over 50,000 homologs of OMBBs to explore the evolutionary origins of these proteins (Franklin et al., 2018a).

The results are surprising. Rather than growing linearly from eight strands to 12 or more, the evolutionary road appears to have been replete with forks and alternate paths. Instead, transitions occur through unexpected mechanisms such as loop-to-strand conversions or the duplication of certain protein domains. In related work, the same team recently reported that certain classes of OMBBs, specifically lysins and efflux pumps, evolved independently of the remainder of OMBBs (Franklin et al., 2018b). Taken together, these results suggest that the emergence of OMBBs with large barrels happened through multiple pathways (Figure 1).

**Figure 1.**
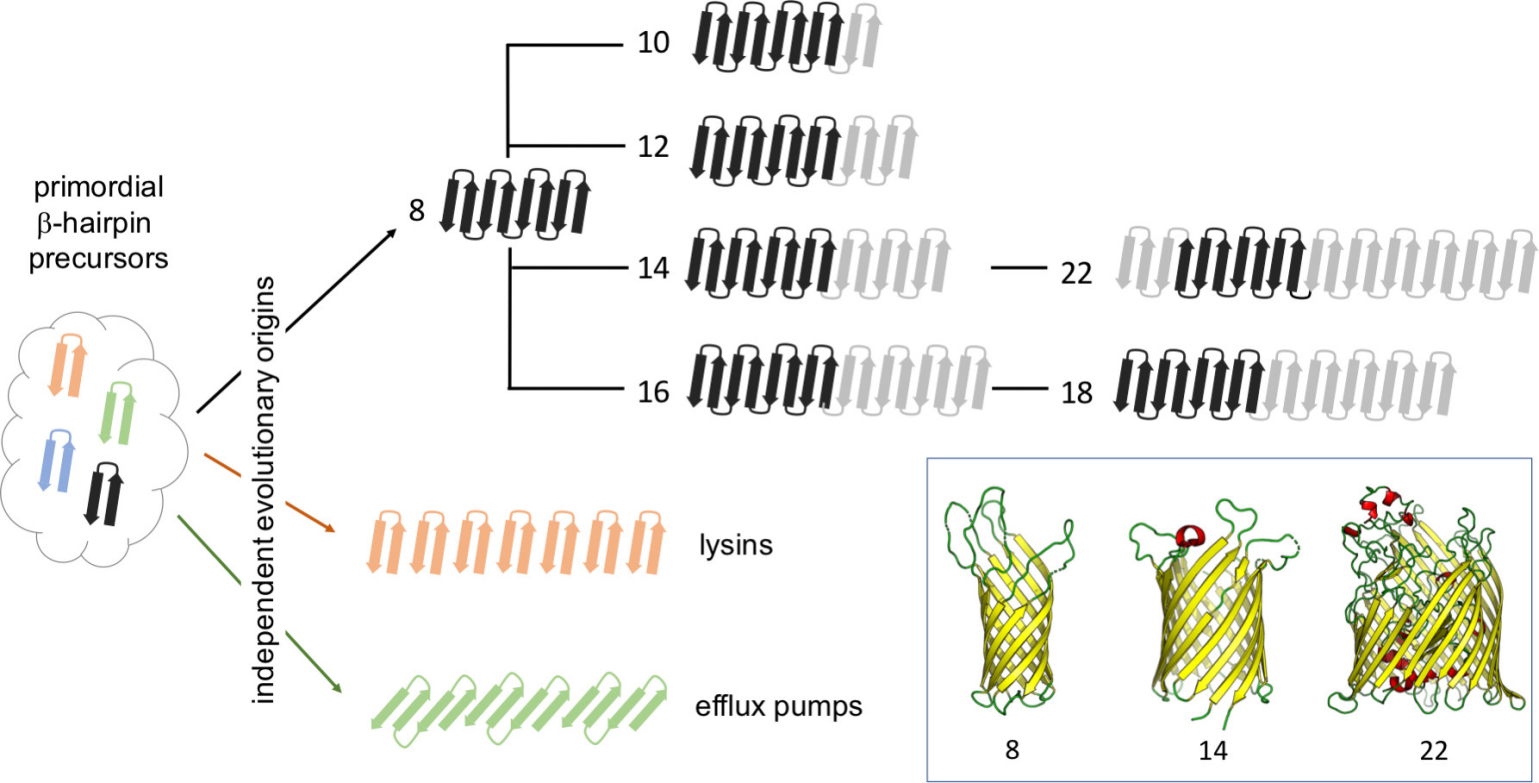
The evolutionary history of beta-barrels. Starting from ancestral pools of β-hairpins, each made of two beta strands (left), the evolution of outer-membrane beta-barrels (OMBBs) containing between eight and 26 strands is thought to have occurred independently multiple times. Beta-barrels containing 10 or more strands evolved as a result of a hairpin duplication at the N-terminus of eight-strand barrels, and it is thought that the folding of these proteins occurs at the C-terminus. The strands in OMBBs form a single continuous chain, and the dark black arrows show strands that span the membrane in which the beta-barrel is embedded; OMBBs with 24 and 26 strands are not shown. Lysins and efflux pumps are examples of multi-chain beta-barrels that evolved independently of single-chain OMBBs and of each other. The inset shows the three-dimensional structures of OMBBs with eight, 14 and 22 beta strands.

Another long-standing puzzle is how OMBBs fold into their final three-dimensional structure. OMBBs face challenges on two fronts: the constraints that apply to the folding of all repeat proteins (Björklund et al., 2006; Wright et al., 2005); and the need to coordinate folding with the insertion of the protein into a membrane (Fleming, 2014). Franklin et al. noted that elements of the original eight-strand sequence have persisted at the C-terminus of larger barrels, suggesting that this region might be responsible for the initial stages of protein folding. Moreover, previous efforts to design even modest variants of an eight-strand barrel were largely unsuccessful (Stapleton et al., 2015): this is consistent with the eight-strand barrels that are observed in nature evolving to have a central role in the folding of OMBBs. This suggests that the best way to engineer larger barrels is to focus on adding new strands to the N-terminus of an eight-strand OMBB.

In exposing the complex evolutionary history of OMBBs, Franklin et al. have given us insights into how nature builds complex proteins from simpler parts. Hopefully, coopers working at the molecular scale can learn from natural evolution and identify new rules for successfully engineering synthetic barrels.
